# Effect of Urea and Borate Plasticizers on Rheological Response of Corn Starch

**DOI:** 10.3390/polym9090361

**Published:** 2017-09-06

**Authors:** Khalid A. Ibrahim, Muhammad Y. Naz, Shaharin A. Sulaiman, Abdul Ghaffar, Yasir Jamil, Nasser M. Abdel-Salam

**Affiliations:** 1 College of Engineering, Muzahimiyah Branch, King Saud University, Riyadh 11451, Saudi Arabia; kibrahim@ksu.edu.sa; 2 Department of Chemical Engineering, Faculty of Engineering, Al-Hussein Bin Talal University, Ma’an 71111, Jordan; 3 Department of Physics, University of Agriculture, Faisalabad 38040, Pakistan; aghaffar16@uaf.edu.pk (A.G.); yasirjamil@uaf.edu.pk (Y.J.); 4 Department of Mechanical Engineering, Universiti Teknologi Petronas, Seri Iskandar 32610, Malaysia; shaharin@utp.edu.my; 5 Arriyadh Community College, King Saud University, Arriyadh 11437, Saudi Arabia; nelsalam@ksu.edu.sa

**Keywords:** carbohydrate polymers, urea, borate, rheology

## Abstract

Although starch based materials have an array of fascinating industrial applications, the native starches do not show good mechanical strength, thermal stability, and rheological properties for their use in the mainstream processing industry. For example, the use of starches for producing controlled release fertilizers is a new research endeavor with detailed knowledge still to come. The thermal processing of native starches with water as a plasticizer results in poor physical and pasting properties of the final product. Therefore in this study, corn starch was thermally processed with urea and borate in a water medium. The pure starch (PS), starch-urea (SU), starch-borate (SB), and starch-urea-borate (SUB) samples were prepared and characterized for their rheological traits. The PS sample exhibited a peak viscosity of 299 cP after 17 min of thermal processing. Further heating of the suspension caused a decrease in viscosity of 38 points due to thermal cracking of the starch granules. A similar trend was depicted in the viscosity measurements of SU, SB, and SUB adhesives. However, the viscosity of these samples remained slightly higher than that for PS. Also, the reduction in viscosity after the peak value was not as notable as for PS. The modified starch behaved like a gel and its storage modulus was significantly higher than the loss modulus. The lower magnitudes of storage and loss moduli revealed that the modified starch was in the form of a weak gel and not a solid. The PS is more fluid in nature with dominating loss modulus at lower angular frequencies.

## 1. Introduction

Starch is a tasteless white-colored carbohydrate powder, which occurs in the form of amylose and amylopectin in tubers, seeds, and other parts of plants. Amylose, a linear polymer, is the simplest form of starch while amylopectin is its branched form. Applications of pure and modified starches have been reported by many researchers during the past few decades [1,2]. The starches are generated from water and carbon dioxide through photosynthesis in plants [3,4]. Native starches are known for low cost, complete biodegradability, and renewability, and therefore are promising candidates for the production of sustainable materials [5]. In many forms, starches are being used in water folders, gelling agents, emulsions, backing, coatings, thickeners, etc. It has also been reported that starches, in their pure form, do not show the good mechanical strength, thermal stability, and rheological properties, generally required for their use in the mainstream processing industry [6,7]. For example, in the paper binding industry, side-seam adhesives should have viscosity in the range 2–4 Pa·s for a total solid content of 20–22% [8]. Such adhesives are prepared with highly soluble white dextrin or acid treated starches to achieve low viscosity of the suspension. Conversely, the bottom paste adhesives, used to seal the bottom of paper bags, are more viscous than the side-seam adhesives. These adhesives are formed of white dextrin and their viscosity can be as high as 140 Pa·s at room temperature. It reveals that chemical modification of native starches is normally required to achieve the desired physico–chemical traits [9].

Starch derivatives are usually produced by reacting some other components with the hydroxyl groups in the native starch molecules. The modified starch shows notably different physico–chemical properties from the parent starch without compromising the biodegradability [10,11]. Substituting the hydroxyl groups in the starch with other groups is an effective way of preparing starch-based materials for an array of industrial applications. The chemical modifications are carried out by adding some minerals or poly(vinyl acetate) to the native starches and heating them at temperatures below the water boiling point. The hydrophobicity of the starch-based adhesives can be improved by adding a fractional amount of poly(vinyl alcohol) or urea-formaldehyde into the dispersion. In some cases, the adhesives are supplemented with plasticizer for minimization of the brittleness of the final product [12]. For example, Mulder et al. [13] modified starch with lignin to produce slow release coatings. Perez et al. [14] mixed and heated urea with kraft lignin to produce slow release fertilizers. Recently, some literature has also been reported on the development of starch-based polymers for conservation of petrochemical resources, reduction of environmental impact, and some other novel applications [10].

Owing to their unique thermal processing properties, starch-based polymers are much more complex than their conventional counterparts [3]. Multiple physical and chemical reactions, including water diffusion, gelatinization, granular expansion, decomposition, melting, and recrystallization are possible during the thermal processing of the starches. Also, many starch-based polymers show non-miscible character, which reflects their poor mechanical properties. It is worth mentioning that the gelatinization trait of starches is of key importance and closely related to the other traits. Gelatinization refers to the destruction of the crystalline structures of granular starch. It is an irreversible multistage process, which starts with granular swelling followed by the native crystalline melting and molecular solubilization. Good information on gelatinization is explicitly required for better conversion of starches into thermoplastics.

Since the decomposition temperature of starches is reported to be lower than their melting temperature [3], the starch-based polymers cannot be thermally processed in the absence of a plasticizer or gelatinization agent. Numerous plasticizers and additives have been assessed for gelatinizing starches during thermal processing. Water is the most popular plasticizer for cooking starch. However, gelatinization of starches, using water as a plasticizer, produces poor mechanical properties and high brittleness due to fast retrogradation. Therefore, to improve the processing properties and performance of the thermally processed starches, other plasticizers and polyols have been widely used in starch processing. These modifiers include glycerol, glycol, sorbitol, sugars, urea, formamide, acetamide, ethylenebisformamide, ethanolamine, citric acid, etc. [15,16]. In general, the characteristics of the native starches such as gelatinization temperature, viscosity, clarity after cooking, retrogradation, and texture not only depend on the nature and wt % of the modifiers but also on the physical environment provided to the starch processing.

In this study, corn starch was thermally processed with urea and borate. The obtained adhesives were highly viscous and non-Newtonian in character, and therefore tested for their rheological traits. The urea was believed to work as a plasticizer other than the water and to facilitate the movement of polymer chains. The borate ions, produced during the dissociation of di-sodium tetraborate, were assumed to be the reactive species for cross-linking [17]. The cross-linking minimizes the starch sensitivity to temperature and stirring rate, and consequently improves resistance against viscosity losses. Therefore, these ingredients in the starch dispersion may ensure the growth of the physical properties desired for the specific use of the starch based polymers.

## 2. Materials and Methods

### 2.1. Preparation of Modified Starch Samples

The food grade corn starch was purchased from the local market (Faisalabad, Pakistan) and dried under sunlight to removal the moisture content. The plasticizer was 98% de-ionized water whereas the other constituents were urea and borate (Na_2_B_4_O_7_·10H_2_O) of Analytical Reagent (AR) grade (Faisalabad, Pakistan). The starch with degree of substitution of 0.81 was reacted with urea and borate in water medium. The degree of substitution of a polymer is defined as the average number of substituent groups attached with a base unit or a monomeric unit. Six samples of the modified starch were prepared and characterized for their physical and rheological traits. Details of the formulation of theses starch based adhesives are given in Table 1.

Initially, 100 mL of de-ionized water was taken in a beaker. The beaker was placed over a hot plate to heat the water to 85 °C. The solution was stirred with a magnetic bar, immersed in the solution, for uniform mixing and heat transfer. Once the desired temperature was reached, 5 g of the completely dried starch was added to the hot water. For complete gelatinization of the starch, the solution was further heated for 30 min at the same temperature. The pure starch (PS) sample was left to cool overnight at room temperature. For the starch-urea (SU) sample, first 5 g of starch was added to the hot water. The starch dispersion was heated for 10 min for better gelatinization. Thereafter, the solution was supplemented with 1 g of urea and heated further for 20 min to complete the reaction. A similar procedure was repeated to prepare the starch-borate (SB) and starch-urea-borate (SUB) samples by adding 1 g of each urea and borate in the starch dispersion at a processing temperature of 85 °C. The whole procedure was repeated again to modify the starch with 2 g of urea and borate and to study the effect of the modifier weight on the rheological traits.

### 2.2. Properties of Modified Starch Samples

The effect of the modifiers on the properties of the modified starch samples was evaluated using different characterization techniques. The gelatinization time, viscosity, as well as storage modulus and loss modulus of the prepared samples were evaluated as a function of temperature and heating time. The controlled experiments were performed by dividing the samples into two groups: Pure starch control (PS) and experimental group of six modified starch compositions. The reliability of the results of viscosity, surface tension, density and other fluid flow parameters was checked by comparison between the controlled and uncontrolled measurements.

An ORCADA^TM^ operated viscometer (Faisalabad, Pakistan) was used for viscosity measurements. It is a portable viscometer for advanced research for the most required fluid parameters. This fully automated viscometer was capable of measuring the viscosity of adhesives, polymers, stimulation fluids, drilling fluids, completion fluids, and cements at varying temperature, shear rate, shear stress and time. In the present case, the sample temperature and shear rate were varied from 20 to 100 °C and from 0 to 1000 s^−1^, respectively. For high accuracy in the measurements, the test temperature was achieved and maintained for 2 min before each run.

The surface tension measurements were carried out using a spinning drop tensiometer. This tensiometer was suitable for very low to high surface tension measurements. The equipment was fitted with a controlled temperature measuring cell. This removable measuring cell included a water coolable high dynamic measuring drive. Inside the measuring cell, helium gas was used as heat carrier to raise the temperature of the measuring capillary to the required level before recording the surface tension of the sample.

Fourier transform infrared spectroscopy (FTIR) was used to produce infrared spectra and to understand the gelatinization mechanism of the pure and modified starches. FTIR is used to identify the bonds in a sample by producing an infrared absorption spectrum. It is also an effective analytical instrument for detecting functional groups and characterizing bond information. An Anton Paar density meter (Faisalabad, Pakistan) was used to measure the density of the prepared samples under similar conditions set for both viscosity and surface tension measurements. The density measurements were based on the oscillating U-tube principle, which ensured high accuracy of the recorded data.

## 3. Results and Discussion

The temperature at which the starch particles undergo a change in state by forming a gel is called the gelatinization temperature. This temperature provides useful information about the heterogeneity of the starch granules. The past reports reveal that most of the starches gelatinize at temperatures ranging from 80 to 90 °C [17,18,19]. It has also been reported that the processing of starches in this temperature range produces adhesive materials of improved physical properties [17,18,19,20]. However, the gelatinization temperature of a starch strongly depends on the heterogeneity of the crystallites within the starch particles [21,22]. The temperature, used for thermal processing of the corn starch, reflected a high resolution of heterogeneity among the crystallites and varying tightness in the particles’ compactness [23].

**Figure 1:**
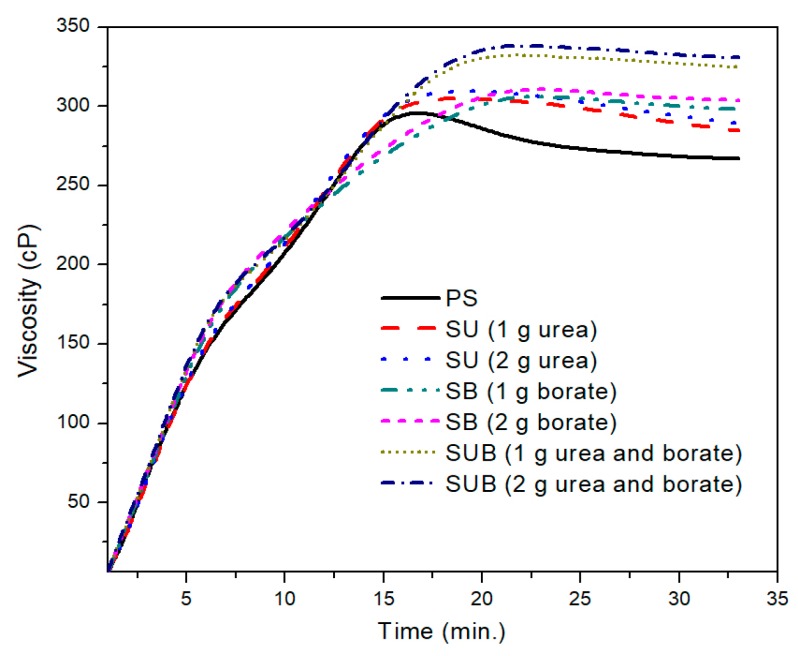
The time-based viscosity plots of PS, SU, SB, and SUB at a fixed processing temperature of 85 °C.

The industrial applications of the starch based materials involve the suspension of the starches in water at temperatures slightly above their gelatinization temperature [23]. Sometimes, the processing conditions imposed on a starch suspension may cause a change in apparent gelatinization temperature and time [24]. Figure 1 shows the time-based viscosity plots of the pure and modified starch samples. It was observed that the urea and borate modifiers together significantly influence the viscosity of the starch based polymers, but for a specific formulation, the amount of the modifiers did not leave a notable impression on the viscosity profiles. For example, a close agreement between the viscosity profiles of the urea modified starch samples (SU), obtained with 1 and 2 g of urea, was noticed in the presented work. A similar trend was reported for SB and SUB compositions. However, a notable difference was observed between the viscosity measurements of SUB and the other compositions (PS, SU and SB).

**Figure 2:**
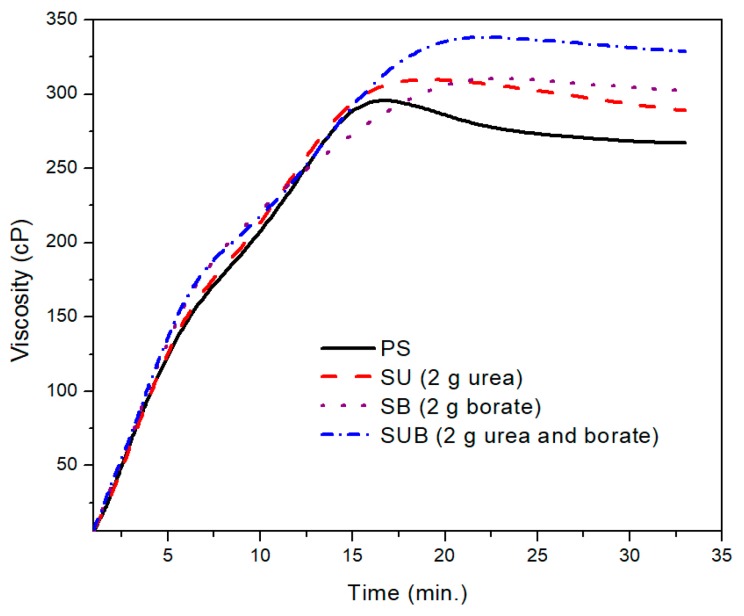
A comparison of the time-based viscosity plots of PS, SU, SB, and SUB adhesives obtained with 2 g of urea and borate.

A further comparison of the PS and those modified with 2 g of the urea and borate is provided in Figure 2. The PS viscosity attained a peak point value after 17 min of heating time at a fixed processing temperature of 85 °C. With further heating of the PS suspension, the viscosity exhibited a slight decrease of 38 points over time. After 27 min of heating time, the viscosity plot reached a constant state and did not vary over time. The peak viscosity of the PS was measured at about 299 cP. A similar trend was depicted in time based viscosity plots of SU, SB, and SUB adhesives. However, the viscosity peaks of these samples were reported slightly higher and the viscosity curves reached their peak values a little latter than the PS viscosity. Also, a reduction in viscosity after the peak point was not as notable as for PS. In the stable region of the viscosity profiles, the viscosities of the PS and SUB samples were measured at about 266 and 330 cP, respectively. It reveals that the viscosity of the starch dispersion was increased by 64 cP after modifying it with small amounts of urea and borate.

**Figure 3:**
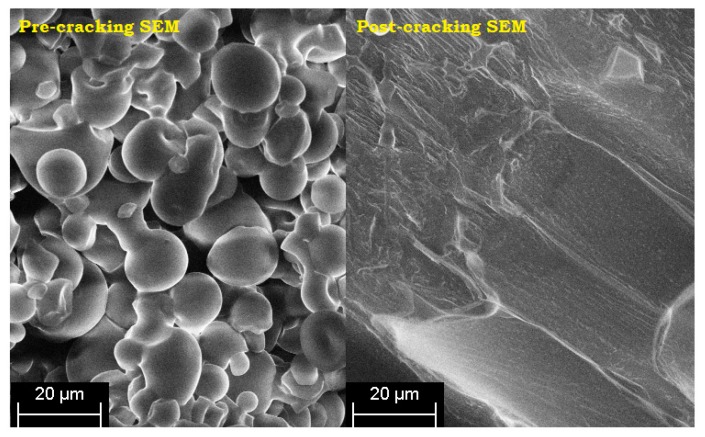
Pre- and post-cracking scanning electron microscopy (SEM) micrographs of pure starch.

**Figure 4:**
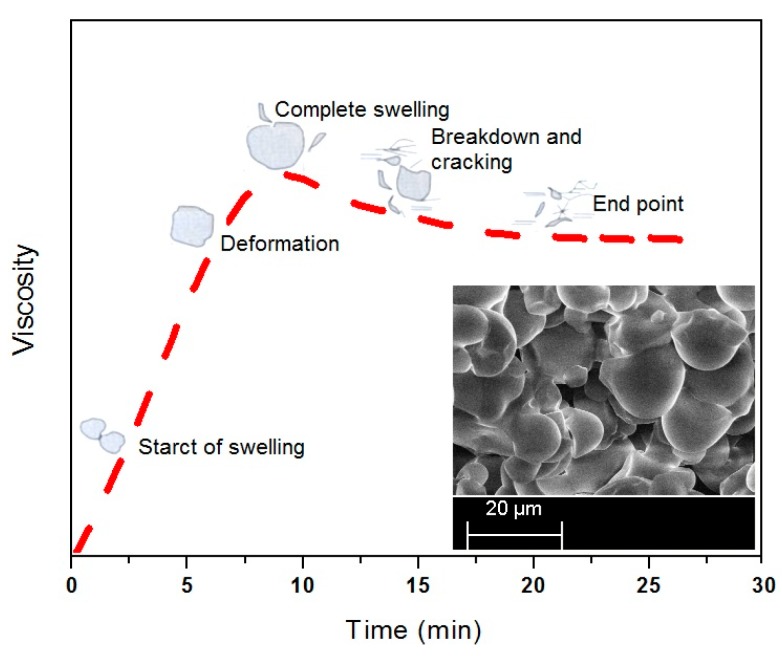
Illustration of time-based swelling of pure starch.

The SEM micrographs of pre- and post-cracking of the corn starch are shown in Figure 3 while Figure 4 illustrates the time-based swelling of the starch particles by using a typical viscosity profile. The alleviation of the PS viscosity after the peak point may be attributed to the reasonably high processing temperature and the extended heating time. As the decomposition temperature of the starch is reported to be lower than its melting point [3], the starch particles quickly expanded over time, as illustrated in Figure 4. Thereafter the particles started to crack by losing their tightness and consequently the adhesive viscosity.

The typical viscosity profile revealed the possibility of change in shape of the particles during thermal processing of the corn starch. The inset provides a photographic view of the thermally processed starch for 30 min. The viscosity breakdown in the time based curve of the pure starch, as suggested in Figure 4, can be prevented or minimized by adding a cross-linking and or plasticizing agent to the starch suspension [25,26]. In Figure 2, a small difference between the peak point and the end point viscosities of the SUB sample suggests that the urea and borate modifiers in the starch suspension significantly reduce the cracking of the starch particles. Therefore, the granules in modified starch suspensions retain their tightness and the breakdown was not as bad as in the case of PS.

A linear increase in viscosity during the early stage of heating is attributed to the swelling and amylose leaching from the starch particles. The viscosity profile attains the peak value after the majority of the particles have swelled out. A further heating of the suspension results in a decrease in viscosity due to break-down of the starch particles under the shear field of the instrument. The mechanical action of the instrument ruptures the swelled particles, in particular the native starch particles. After break-down the amylopectin molecules start to shear and solubilize by reducing the molecular weight of the amylopectin, subsequently, the adhesive viscosity [27,28]. In line with this, the shape of the pasting curve may also depend on the starch type, chemical additives, pH of the suspension, applied shear rate, heating time, temperature, and impurities [29]. The other physical conditions, applied during the starch processing, also influence the gelatinization time and shape of the pasting curve [24].

**Figure 5:**
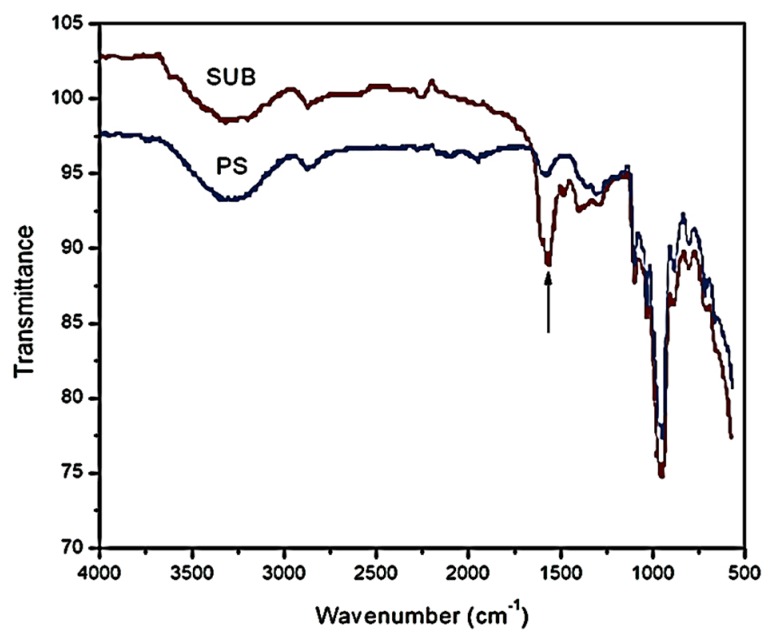
Infrared spectra of PS and SUB samples.

Since modified starch samples revealed an almost similar trend in viscosity profiles, only PS and SUB samples were compared and discussed further. In SUB complex, it is believed that the di-sodium tetraborate molecules dissociate into sodium and borate ions [17]. FTIR technique was used to understand the gelatinization mechanism of the pure and modified starches by producing infrared spectra. Figure 5 shows the FTIR spectra of PS and SUB samples in the range of 500–4000 cm^−1^. A new peak appeared in the FTIR spectrum of the SUB sample at a wavenumber of 1555 cm^−1^. This peak shows the presence of secondary amide in the SUB sample. In a secondary amide, the amido-group (nitrogen) is directly bonded to two carbon atoms. This secondary amide was produced during a polymerization reaction between urea and starch in the presence of borate. In response, molecular weight, density, viscosity and surface tension of the adhesive were improved in the presence of urea and borate in the starch suspension [27].

**Figure 6:**
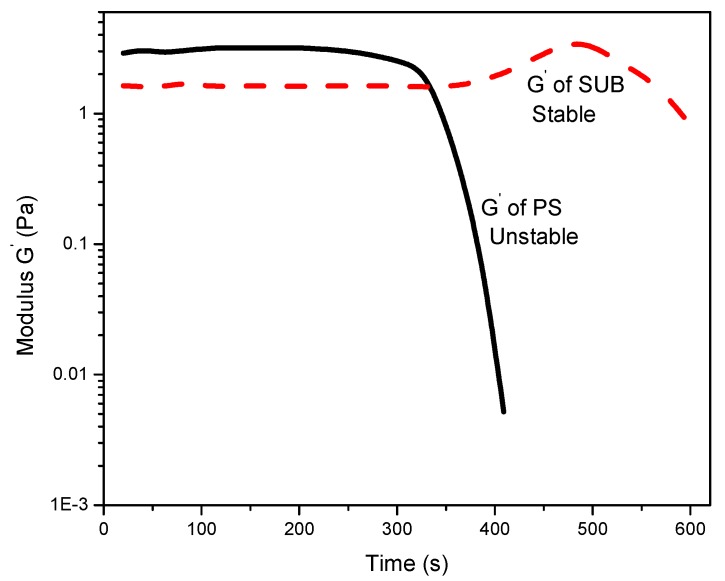
The storage modulus plot of pure and modified starch.

Figure 6 reports the change in storage modulus with time. The modified starch showed good stability whereas PS exhibited poor stability over time. A sharp decrease in storage modulus of PS after 300 s suggests that the gel structure is not stable and breaks down after a certain interval of time. It is also observed that the modified starch retains its gel structure and slightly thickens over time after 380 s. The reason for the apparent thickness of the modified starch gel may be twofold: water evaporation and thixotropy. The tendency of water to evaporate during longer periods of measurements may result in false thickening of the gel. Another possibility is that the solution could be thixotropic, i.e., the behavior changes over time.

**Figure 7:**
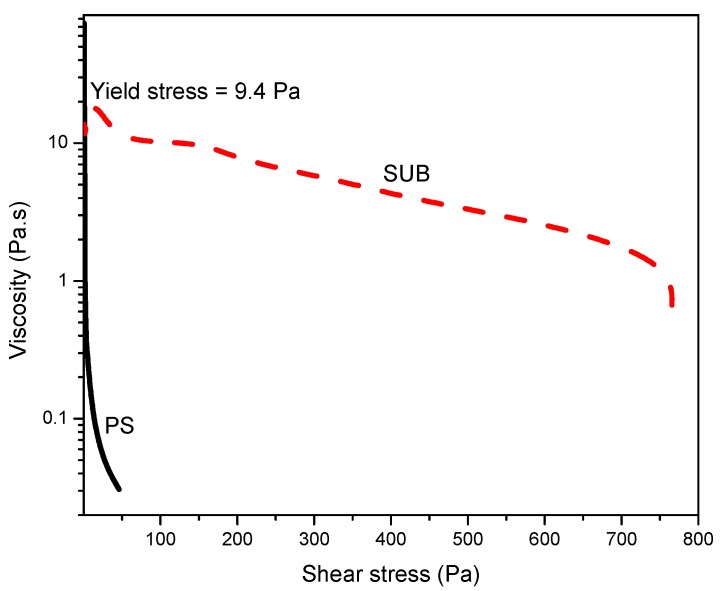
Stress ramp for measurement of yield stress.

The viscosity of the concentrated dispersions, when subjected to the stress or shear rate, does not reach a steady state for some time due to the instability of the internal network structures. The structures, fragmented by the shearing, take time to rebuild. A steady state would appear in the respective viscosity plot soon after establishment of an equilibrium between the breakdown and rebuilding of the structures. Figure 7 shows the stress ramp and yield stress at room temperature. This plot was used to measure the yield stress: the stress value below which the sample behaves like an elastic fluid and which above this value, the sample starts to flow. In this plot, the PS behaved like a fluid which started to flow immediately with the application of stress and SUB behaved like a week gel with a yield stress of 9.4 Pa.

**Figure 8:**
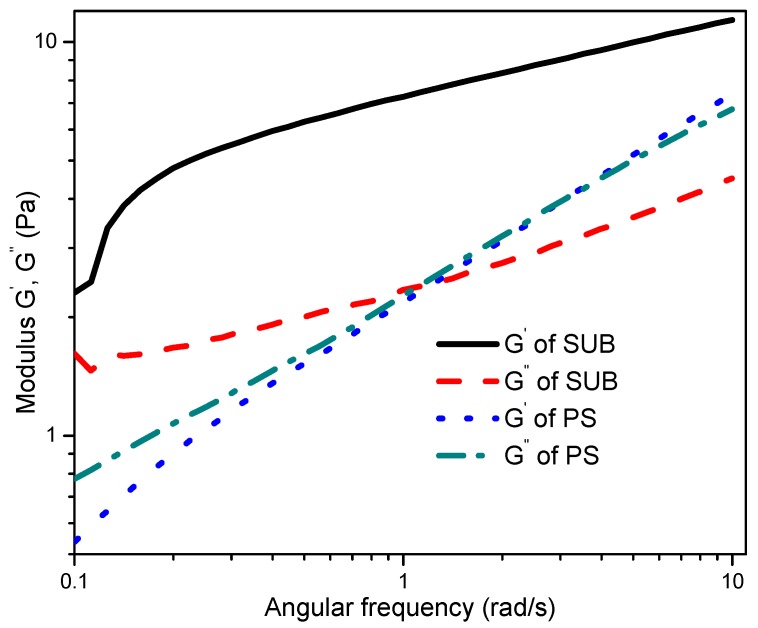
Frequency sweep response of pure and modified starch.

The frequency sweep response of PS and SUB adhesives at 1% strain is compared in Figure 8. The storage and loss moduli of the samples were obtained by changing the angular frequency at room temperature. The modified starch behaved like a gel and its storage modulus was significantly higher than the loss modulus. Conversely, the PS showed dense fluid like character. The gel-like behavior of the SUB sample is attributed to a significantly higher elastic component than a viscous component. However, for a strong gel, the magnitudes of both elastic and viscous components should reach up to a million.

The low magnitudes, as depicted from the frequency sweep curves, indicate that the modified starch was in the form of a weak gel but not a solid. The PS was more fluid in nature with the viscous component being more dominant at the lower angular frequencies. One can say that in spray coating applications, these modified starch samples would produce larger droplets with steady jet breakup. Finally, the elastic and viscous components of the PS showed an almost similar increasing trend over changing angular frequency. Both parameters were in good agreement at higher angular frequencies. The reported literature reveals that thermally processed starches show shear-thickening response when the quantity of the modifier in the dispersion is high enough to cause particle crowding [27,30].

**Figure 9:**
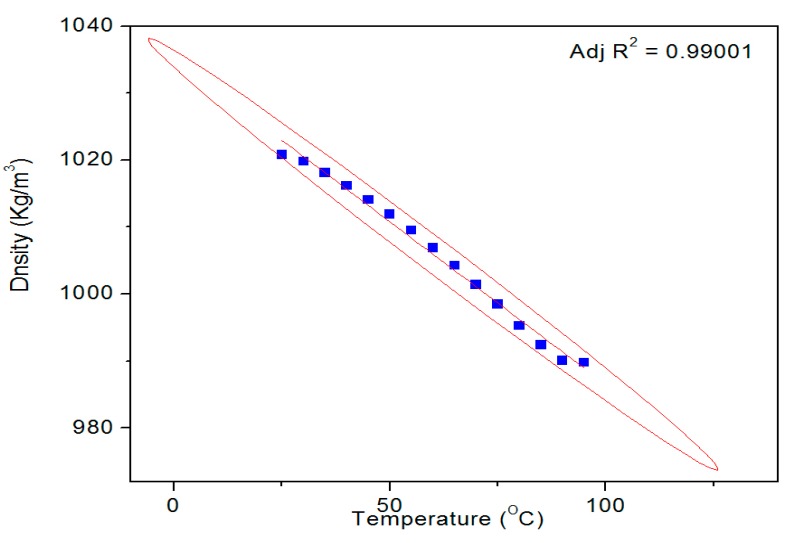
A typical temperature dependent density profile of SUB sample.

Figure 9 shows a typical temperature dependent density profile of the SUB sample. The pure and modified starch samples were also tested for their surface tension and density. The results of these measurements are given in Table 2. Both parameters exhibited slightly decreasing trend with a rise in run temperature from room temperature. The surface tension of SUB remained negligibly higher than PS [27] at all temperatures. Conversely, the density of the SUB sample remained slightly higher than the PS at lower temperatures (30–70 °C) but reached the same values as the PS at higher temperatures. These results show that the modifiers in the dispersion did not significantly influence its surface tension and density.

## 4. Conclusions

The presence of small amounts of urea and borate in a starch dispersion can significantly alter the physical properties of the final product. Urea works as a plasticizer other than water and facilitates the movement of the polymer chains. The unmodified starch attained peak viscosity after 17 min of heating at a fixed temperature of 85 °C. With further heating, the viscosity exhibited a slight decrease of 38 points over time. Although a similar trend was depicted in time based viscosity plots of modified starches, the peak viscosities of these samples were reported to be slightly higher than the pure starch. Also, the reduction in viscosity after the peak value was not as notable as for pure starch. A small difference between the peak point and end point viscosities of the modified starch samples suggested that the urea and borate modifiers significantly reduced the cracking of the starch particles. Therefore, the granules in modified starch suspensions retained their tightness and the breakdown was not as bad as in the case of PS. In the FTIR spectrum of SUB, a new peak appeared at a wavenumber of 1555 cm^−1^. This peak showed the presence of secondary amide, which was produced during the polymerization reaction between urea and starch in the presence of borate. In response, molecular weight, density, viscosity, and surface tension of the SUB adhesive were improved in the presence of urea and borate in the starch suspension.

The modified starches showed good stability whereas pure starch exhibited poor stability over time. A sharp decrease in storage modulus of pure starch after 300 s suggested an unstable gel structure, which breaks down after a certain interval of time. The modified starches behaved like weak gels and their storage modulus was significantly higher than the loss modulus. Contrarily, the pure starch showed dense fluid like character. The lower magnitudes, as depicted from the frequency sweep curves, revealed that the modified starch was in the form of a weak gel and not a solid. The pure starch was more fluid in nature with the viscous component being more dominant at lower angular frequencies.

## Figures and Tables

**Table 1:** Composition of the modified starch samples described below.

Sample	Water (mL)	Starch (g)	Urea (g)	Borate (g)
PS	100	5	-	-
SU	100	5	1	-
SU	100	5	2	-
SB	100	5	-	1
SB	100	5	-	2
SUB	100	5	1	1
SUB	100	5	2	2

**Table 2:** Density and surface tension of the thermally processed starch.

Temp. (°C)	Density (Kg/m^3^)		Surface Tension (mN/m)	
	PS Sample	SUB Sample	PS Sample	SUB Sample
30	1017	1019	68.7	69.0
40	1013	1016	68.6	68.9
50	1009	1011	68.4	68.5
60	1004	1005	68.0	68.2
70	999	1000	67.7	67.8
80	993	993	67.2	67.4
90	990	990	67.0	67.1
